# The implications of abnormal signal patterns of break-apart FISH probes used in the diagnosis of bone and soft tissue tumours

**DOI:** 10.3389/pore.2025.1612142

**Published:** 2025-07-01

**Authors:** Hongtao Ye, Fitim Berisha, Evie Rowles, Emani Munasinghe, Christopher Davies, Akanksha Farswan, Nischalan Pillay

**Affiliations:** ^1^ Department of Cellular and Molecular Pathology, Royal National Orthopaedic Hospital, Stanmore, United Kingdom; ^2^ Cancer Institute, University College London, London, United Kingdom

**Keywords:** FISH, abnormal signal pattern, break-apart probe, bone and soft tissue tumour, gene rearrangement, translocation, cancer, genomics

## Abstract

Many subtypes of bone and soft tissue tumours harbour specific chromosome translocations leading to chimeric fusion genes. The identification of these specific fusion genes is the basis of molecular diagnoses in such tumours. Break-apart FISH is a robust method that is commonly used to identify these translocations and provide diagnostic support to histological interpretations. The signal patterns of the break-apart probes are usually easily interpreted. However, some cases show abnormal signal patterns leading to equivocal and challenging interpretation. The incidence of these abnormal patterns is largely unknown. Using a retrospective cohort we explored the incidence of abnormal signal patterns across common bone and soft tissue tumour types to raise awareness of this occurrence and to aid in the interpretation. In total, 1,087 bone and soft tissue tumours tested by break-apart probes were examined. The abnormal signal patterns were classified as deletion, additional copy and amplification, which were found at highest frequency in low-grade fibromyxoid sarcoma (32%, 6/19), and at moderate frequencies in those from alveolar rhabdomyosarcoma (10%, 9/94), nodular fasciitis (9%, 18/209), synovial sarcoma (8%, 17/207) and Ewing sarcoma/round cell sarcoma with EWSR1-non-ETS fusions (6%, 29/497). The lowest frequency was found in clear cell sarcoma (1%, 1/61). Despite the equivocal results from the abnormal signal patterns, the specific fusion genes were confirmed by orthogonal molecular techniques such as FISH with fusion probes, RT-PCR or next-generation sequencing.

## Introduction

The accurate diagnosis and subsequent clinical management of many bone and soft tissue tumours are reliant on molecular testing. Many of these tumours have characteristic genetic translocations (Table 1) [1–4], which can be detected by fluorescence *in situ* hybridization (FISH), reverse transcription polymerase chain reaction (RT-PCR) and Next-Generation Sequencing (NGS).

**TABLE 1:** Chromosome translocation in bone and soft tissue tumours.

Type of tumour	Chromosome abnormality	Gene involved	Frequency	Break-apart probe
Alveolar rhabdomyosarcoma Clear cell sarcoma Ewing sarcoma/PNET Round cell sarcoma with EWSR1-non-ETS fusions Low grade fibromyxoid sarcoma Nodular fasciitis Synovial sarcoma	t (2; 13) (q35; q14) t (1; 13) (p36:q14) t (12; 22) (q13; q12) t (2; 22) (q34; q12) t (10; 22) (p11; q12) t (11; 22) (q24; q12) t (21; 22) (q22; q12) t (20; 22) (q13; q12) t (7; 16 (q34; p11) t (11; 16) (p11; p11) t (17; 22) (p13; q13) t (x; 18) (p11; q11)	*PAX3::FOXO1* *PAX7::FOXO1* *EWSR1::ATF1* *EWSR1::CREB1* *EWSR1::CREM* *EWSR1::FLI1* *EWSR1::ERG* *EWSR1::NFATC2* *FUS::CREB3L2* *FUS::CREB3L1* *MYH9::USP6* *SS18::SSX1* *SS18::SSX2* *SS18::SSX4*	85% 10% 90% 10% rare 85% 10% <1% 90% 5% 90% 90% 10% rare	FOXO1 EWSR1 EWSR1 FUS USP6 SS18

The signal patterns of the break-apart probes are usually easily interpreted. However, some cases may show abnormal signal patterns leading to challenging interpretation, equivocal results and therefore uncertainty in diagnosis and management. Several studies have systemic examined abnormal signal patterns in bone and soft tissue tumours [5–7], however, the true incidence and significance of the abnormal signal patterns in different subtypes are unknown.

The present study aims to explore the incidence of abnormal signal patterns by tumour type to raise awareness of this occurrence and correlate with genomic sequencing where available.

## Materials and methods

A total of 1,087 samples were retrieved from our files in the Department of Cellular and Molecular Pathology, Royal National Orthopaedic Hospital (Table 2). They included 94 cases of alveolar rhabdomyosarcoma (ARMS), 61 cases of clear cell sarcoma (CCS), 497 cases of Ewing sarcoma/round cell sarcoma with EWSR1-non-ETS fusions, 19 cases of low-grade fibromyxoid sarcoma (LGFMS), 209 cases of nodular fasciitis (NF) and 207 cases of synovial sarcoma (SS). All cases were classified according to the World Health Organization classification (WHO) of bone and soft tissue tumours [1]. All cases analysed by FISH using break-apart probes were reviewed to identify the frequency and type of abnormal signal patterns. These were classified as deletion, amplification, and extra copy of the gene locus.

**TABLE 2:** Abnormal signal pattern of break-apart probe and fusion gene detected.

Type	ARMS (FOXO1)	CCS (EWSR1)	ES/RCSEEF	LGFMS (FUS)	NF (USP6)	SS (SS18)
No. of cases	94	61	497	19	**209**	**207**
Deletion	0	0	8 (2%)	0	16 (8%)	8 (4%)
Extra copy	0	1 (2%)	15 (3%)	6 (32%)	2 (1%)	9 (4%)
Amplification	9 (10%)	0	6 (1%)	0	0	0
Fusion gene detected by FISH^a^/RT-PCR^a^/NGS^b^/WGS^b^	3/9 *PAX3::FOXO1* (+)^a^ 6/9 *PAX7::FOXO1* (+)^a^	1/61 *EWSR1::CREM* (+)^b^	12/29 *EWSR1::FLI1* (+)^a^ ^,^ ^b^ 9/29 *EWSR1::ERG* (+)^a^ ^,^ ^b^ 6/29 *EWSR1:NFATC2* (+)^a^ ^,^ ^b^ 1/29 *EWSR1::FLI1(ERG)* (−)^a^ 1 case no material available	4/6 *FUS::CREB3L2* (+)^a^ 2/6 *FUS::CREB3L2* (−)^a^	11/18 *MYH9::USP6* (+)^a^ ^,^ ^b^ 1/18 *FRMD6::USP6* (+)^b^ 5/18 *MYH9::USP6* (−)^a^ 1 case no material available	13/17 *SS18::SSX1* (+)^a^ ^,^ ^b^ 3/17 *SS18::SSX2* (+)^a^ ^,^ ^b^ 1 case no material available

Note: ARMS, alveolar rhabdomyosarcoma; CCS, clear cell sarcoma; ES, ewing sarcoma; RCSEEF, round cell sarcoma with EWSR1-non-ETS fusion; LGFMS, low grade fibromyxoid sarcoma; NF, nodular fasciitis; SS, synovial sarcoma.

^a^ FISH/RT-PCR assay.

^b^ NGS, Next-Generation Sequencing/WGS, whole genome sequencing.

### Fluorescence *in situ* hybridization (FISH)

FISH analysis was performed on formalin fixed paraffin embedded sections using dual color break-apart probes, namely EWSR1, FOXO1, FUS, SS18 (Abbott Molecular, USA), NFATC2 (Agilent Technologies, California, USA) and USP6 (ZytoVision, Bremerhaven, Germany). Fusion FISH probes of PAX3::FOXO1, PAX7::FOXO1, EWSR1::FLI1 (ZytoVision, Germany) and EWSR1::NFATC2 (Wuhan Kanglu Biotechnology Co., Ltd, China) were used as an alternative method to confirm the translocation in a spectrum of equivocal cases. The procedure of FISH assay was described previously [8]. After dewaxing in xylene and rehydration in a series of ethanol, sample sections were cooked in a pressure cooker for 5 min in deionized water. Then the sections were digested in 0.125% pepsin solution at 37°C for 50 min. The specific break-apart probe was co-denatured on the sections at 72°C for 15 min and hybridized at 45°C overnight in a humidified box in an oven. Post hybridization washings were carried out and counterstained with 4.’6′- diamidino-2-phenylindole from Vector Laboratories (Burlingame, CA, United States). Fifty non-overlapping nuclei were counted for each case. The typical positive signal pattern was interpreted as 1 yellow, 1 red and 1 green and if the green and a red signal were separated by more than two times distance of the size of one signal in more than 15% of the counted cells. The abnormal signal patterns were classified as a deletion, amplification, and extra copy of the gene locus. When 1 yellow and 1 to a few green signals without a red signal/or 1 yellow and 1 to a few red signals without green signal were considered as a deletion. If the number of green/or red signals were too numerous to be counted it was considered as an amplification. If 2 or more yellows and 1 to few greens without red signal or 2 or more yellows and 1 to few reds without a green signal were considered as an extra copy of the gene. An abnormal signal pattern is considered if more than 15% counted cells showing same abnormal signal pattern.

### RNA extraction and reverse transcription

RNA was extracted from 10 µm sections cut from paraffin-embedded resection or biopsy samples. FFPE Ambion Recoverall Total Nucleic Acid isolation kit (Invitrogen, Thermo Fisher Scientific, United States). Between 1 and 3 µL of the RNA samples were reverse transcribed using Superscript III First-Strand Synthesis kit (Invitrogen, Thermo Fisher Scientific, United States) using random hexamers. All steps were performed according to the manufacturer instructions.

### Conventional polymerase chain reaction

PCR amplification was performed on duplicate samples of 1 µL aliquots of cDNA using specific primer sets designed based on the known fusion genes and break points (all primers and product sizes shown in Table 3). Reactions were performed in 25 µL using 1 × buffer II, 200 µM of each dNTP, 5 pmol of each primer, 1.5 mM MgCI_2_, and 1 U of Platinum Taq DNA polymerase (Applied biosystems, Thermo Fisher Scientific, United States). A touchdown protocol was used with cycling parameters as follows: 7 min at 95°C followed by 45 s at 94°C, 45 s at 66°C, 1 min 30 s at 72°C which was followed by reducing the annealing temperature by 1°C each cycle to 57°C (10 cycles), followed by 30 cycles at 56°C and finally 5 min at 72°C [5].

**TABLE 3:** Primers and expected product sizes of RT-PCR.

Strand primer sequence 5’ – 3′	Product size (bp)
G6PD primers G6PD 86 Sense ACGGCAACAGATACAAGAAC G6PD 141 Sense CCAAGAAGCCGGGCATGT G6PD 200 Sense GCGCAACGAGCTGGTGAT G6PD Anti-sense CGAAGTGCATCTGGCTCC EWSR1 exon7 Sense CTGGATCCTACAGCCAAGCTCCAAG FLI1 exon6 Anti-sense GTTGAGGCCAGAATTCATGTTA ERG exon7 Anti-sense ACCGGTCCAGGCTGATCT ERG exon10 Anti-sense AACTGCCAAAGCTGGATCTG FUS exon6 Sense GCTATGAACCCAGAGGTCGT CREB3L2 exon5 Anti-sense TTATGAGGAGCCGTGAGGAG MYH9 exon1 Sense GGGGCAGATCCAGGTTCAG USP6 exon1 Anti-sense GAAACTGGGCATCTCTGTGGC USP6 exon2 Anti-sense GATGGACATGGTAGAGAATGC PAX3 Sense CCGACAGCAGCTCTGCCTAC PAX7 Sense CCGACAGCAGCTCTGCCTAC FOXO1 Anti-sense TGAACTTGCTGTGTAGGGACAG SS18 Sense AGACCAACACAGCCTGGACCAC SSX1 Anti-sense ACACTCCCTTCGAATCATTTTCG SSX2 Anti-sense GCACTTCCTCCGAATCATTTC	86 141 200 Type I: 125, type II: 191 94 81 64 to 121 208 173 169 160 108 108

PCR products were analyzed by electrophoresis on 8% polyacrylamide gels, stained with GelRed and visualized under UV illumination using a Syngene NuGenius Gel imaging system (Cambridge, UK). Samples yielding PCR products of the predicted size in both reactions were considered as positive. A negative (no template) and positive controls of the specific fusion genes confirmed by sequencing were used for each experiment.

The housekeeping gene of glucose-6-phosphase dehydrogenase (*G6PD*) was amplified in parallel using the same reaction conditions. The PCR primers were designed to provide the template for generation of products of 86, 141 and 200 bp, which are the controls for RNA quality of the samples.

### Next-generation sequencing (NGS)

FFPE samples were referred to a centralized Genomic Laboratory Hub to perform targeted RNA sequencing (RNAseq).

### Whole-genome sequencing

High quality DNA was extracted from frozen tissue using QIAamp DNA mini kit (Qiagen, Hilden, Germany). Both tumour and matching normal samples were sequenced using the Novoseq (Illumina Inc.) using a PCR free workflow.

The tumour samples were sequenced to a depth of 70X and normal/germline to 30X. Fastq files were QC’ed, aligned and pre-processed using bcbio-nextgen pipeline [9]. The three SV callers – Manta [10], GRIDSS2 [11], and Svaba [12] were used to determine the structural variants (SVs), and the final agreement was reached by considering calls from at least two out of three callers.

## Results

**FIGURE 1:**
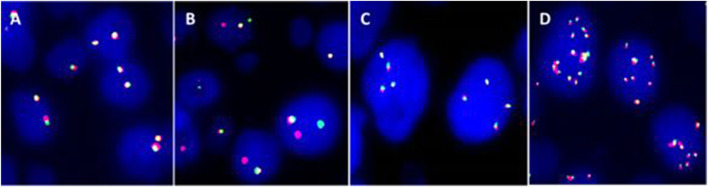
Usual signal pattern of break-apart probe. **(A)** Negative signal pattern of two yellow signals. **(B)** Positive signal pattern of one yellow and a break-apart (one green separated from one red). **(C)** Negative: Three to 4 copies of yellow signals. **(D)** Negative: Multi-copies of yellow signals.

**FIGURE 2:**
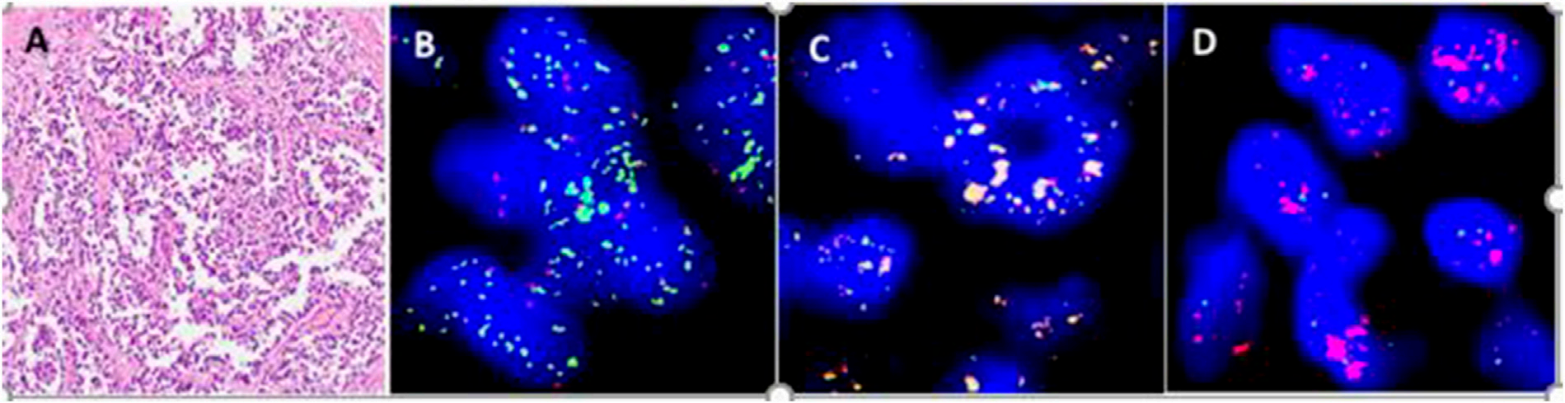
An alveolar rhabdomyosarcoma with *FOXO1* gene amplification. **(A)** Round cells with pseudoalveolar pattern. **(B)** *FOXO1* break-apart probe shows positive signal pattern with amplification of 3′ end of the *FOXO1* locus (Green). **(C)** Multi fusion yellow signals of *PAX7::FOXO1* by FISH of PAX7::FOXO1 fusion probe. **(D)** No fusion signal found by FISH of *PAX3::FOXO1* fusion probe.

**FIGURE 3:**
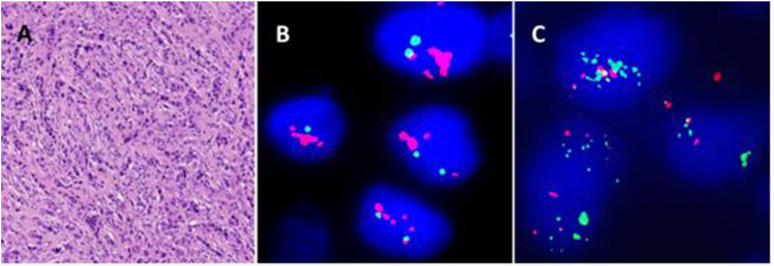
A *NFATC2*-rearranged sarcoma with *EWSR1* gene amplification. **(A)** Undifferentiated blue small round cells composed of cords of cells in a fibrous stroma. **(B)** *EWSR1* break-apart probe shows atypical signal pattern: amplification of 5′ end of the *EWSR1* locus. **(C)** NFATC2 break-apart probe confirmed *NFATC2* gene rearranged with amplification of 3′ end of the *NFATC2* locus. *EWSR1::NFATC2* fusion gene detected by NGS.

**FIGURE 4:**
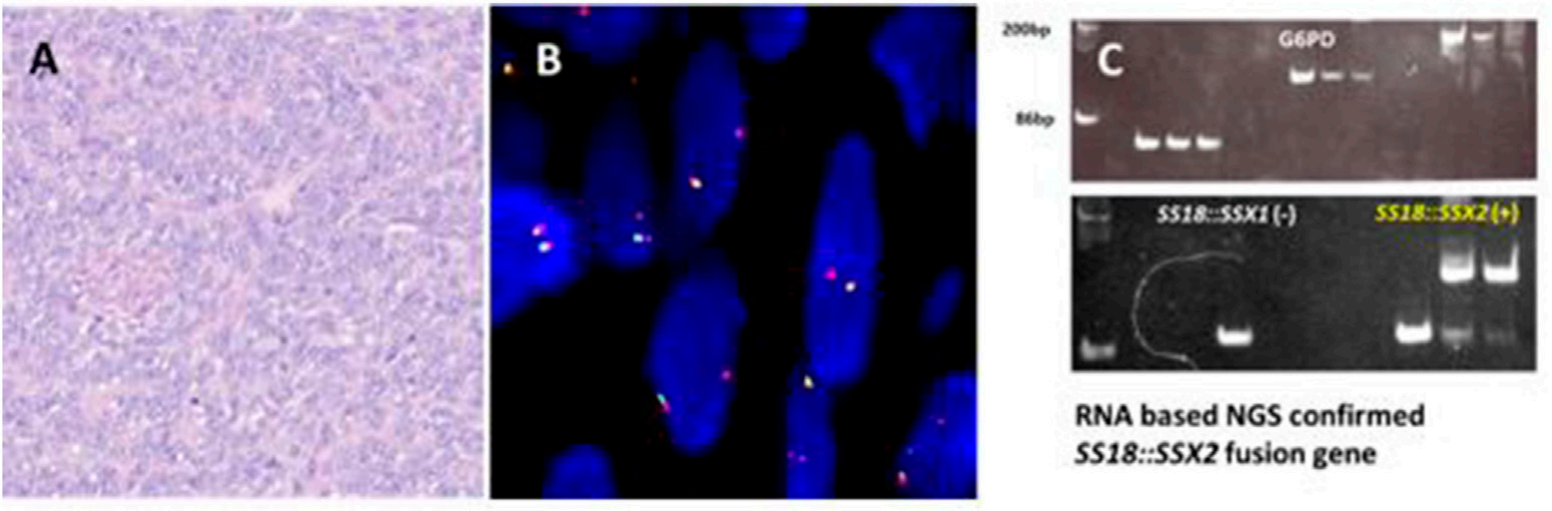
A synovial sarcoma with deletion of 3′ end of *SS18* locus. **(A)** Monophasic-type SS. **(B)** *SS18* break-apart probe shows atypical signal pattern of one yellow and one red signals: deletion of 3′ end of the *SS18* locus. **(C)** *SS18::SSX2* fusion gene detected by RT-PCR and NGS.

**FIGURE 5:**
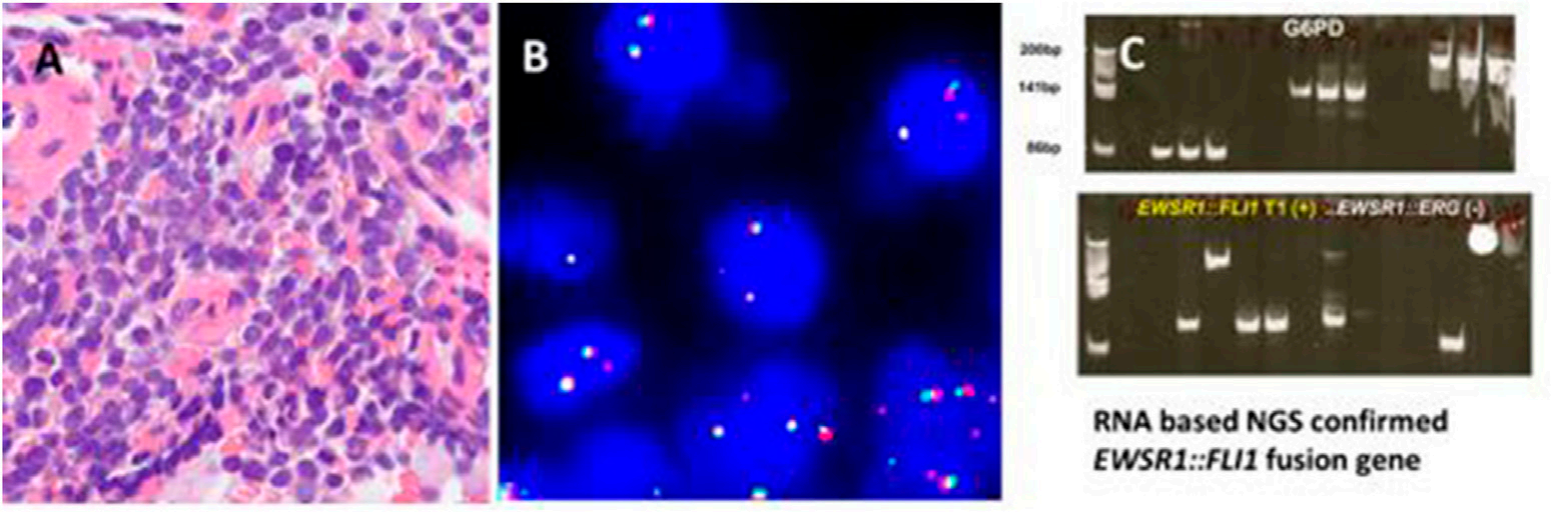
An Ewing sarcoma with no break-apart and an extra copy of *EWSR1* gene locus. **(A)** Monomorphic small blue round cell tumour. **(B)** *EWSR1* break-apart probe shows atypical signal pattern of two yellow and one red signals: one extra copy of the 5′ end of the *EWSR1* locus. **(C)** *EWSR1::FLI1* type I fusion gene detected by RT-PCR and NGS.

**FIGURE 6:**
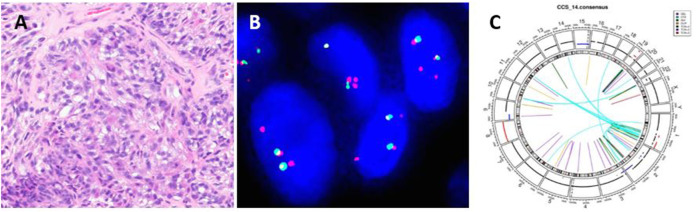
A clear cell sarcoma with extra copies of 5′ end of *EWSR1* locus. **(A)** Polygonal cells with vesicular nuclei and cytoplasmic clearing forming sheets and nests. **(B)** *EWSR1* break-apart probe shows atypical signal pattern of two yellow and two red signals: extra copy of 5′ end of the *EWSR1* locus. **(C)** Circos plot of *EWSR1::CREM* fusion gene detected by WGS.

Break-apart FISH analysis was performed in 1,087 cases demonstrating informative results in Table 2; Figures 1–6, including 94 ARMS, 61 CCS, 497 Ewing sarcoma/round cell sarcoma with EWSR1-non-ETS fusions, 19 LGFMS, 209 NF and 207 SS. The summary of abnormal signal patterns is shown in Table 2. Abnormal signal patterns were detected in 7% (80/1,087 cases), including 10% (9/94 cases) of ARMS (*FOXO1*) (Figure 2), 1% (1/61 cases) of CCS (*EWSR1*) (Figure 6), 6% (29/497 cases) of Ewing sarcoma/round cell sarcoma with EWSR1-non-ETS fusions (*EWSR1*) (Figures 3, 5, respectively), 32% (6/19 cases) of LGFMS (*FUS*), 9% (18/209 cases) of NF (*USP6*) and 8% (17/207 cases) of SS (*SS18*) (Figure 4).

The typical pattern of break-apart probe signals in an interphase nucleus without a gene rearrangement are two yellow signals (red/green overlapping signals) which represent two normal gene loci (Figure 1A). A signal pattern consisting of one yellow signal, split signal pattern of one red signal and a separated green signal in a nucleus indicates a typical positive signal pattern (Figure 1B). Multiple yellow signals without separate red and green signals are considered as negative (Figures 1C,D).

94 putative ARMS were screened by FOXO1 break-apart probe for *FOXO1* gene rearrangement (Figure 2A). The average age of ARMS patients was 30 years (range 1–85 years) with a 1:1 male to female ratio. The most common site of occurrence was the extremities (25, 27%) and nasal cavity (20, 21%). Most of cases displayed the split pattern of separate red and green signals, however, 9 cases demonstrated FOXO1 amplification with few yellow signals, 1 to 2 red and amplification of 3′ of the *FOXO1* locus of more than 15 copies of green signals (Figure 2B). Three of 9 cases carrying the *PAX3::FOXO1* fusion gene were confirmed by PAX3::FOXO1 fusion probe. *PAX7::FOXO1* fusion gene was detected in six of 9 cases by *PAX7::FOXO1* fusion probe (Table 2; Figures 2C,D). Among these 9 cases with an amplification signal pattern, the majority of cases were female (6). Six of 9 cases were from the extremities. No deletion or extra copy of *FOXO1* locus was found in any of the 94 cases of alveolar rhabdomyosarcomas screened.

61 CCS were screened for an *EWSR1* gene rearrangement (Figure 6A). The average age of CCS patients was 34 years (range 9–73 years) with a 1:1.6 male to female ratio. The most common site of occurrence was the distal lower extremities (39, 64%). Nearly all cases showed the typical positive signal pattern of 1 yellow, 1 red and 1 separated green except one case (2%, 1/61 cases) which displayed an extra copy of *EWSR1* locus with 2 yellow and 2 red signals (Figure 6B). This case demonstrated the rare *EWSR1::CREM* fusion which was identified by both RNAseq NGS and WGS (Figure 6C). No deletion or amplification of *EWSR1* locus was found in any of the clear cell sarcoma cases assessed.

A total of 497 Ewing sarcoma/round cell sarcoma with EWSR1-non-ETS fusions were tested for the *EWSR1* gene rearrangement (Table 2). The average age of ES/round cell sarcoma with EWSR1-non-ETS fusions patients was 23 years (range 1–87 years) with a 1.7:1 male to female ratio. 57% of the patients were <20 years old at diagnosis. The most common sites of involvement were long bone in 40% (199/497), followed by pelvis 16% (81/497) and ribs 8% (40/497). Abnormal signal patterns were detected in 29 (6%, 29/497) cases. These included 8 cases (2%, 8/497) which showed a deletion pattern with 1 yellow and 1 to 2 red signals of 5′ end of the *EWSR1* locus, 15 cases (3%, 15/497) with an extra copy with 2 yellow and 1 to 4 red signals (Figure 5), and 6 cases (1%, 6/497) showing 1 yellow, 1 green and amplification of 5′ end of the *EWSR1* locus with more than 8 to 20 copies of red signals (Figure 3). Among 8 cases displaying the deletion signal pattern, the *EWSR1::FLI1* fusion was proved in 5 cases by FISH, RT-PCR or NGS, whilst the *EWSR1::ERG* fusion gene was identified in 2 cases. One case had no remaining material available for further testing. In the 15 cases showing extra copy abnormal signal patterns, the *EWSR1::FLI1* fusion gene was detected in 7 cases by FISH, RT-PCR or NGS (Figure 5). The *EWSR1::ERG* fusion gene was found in 7 cases by RT-PCR or NGS. The *EWSR1::NFATC2* fusion gene was detected by FISH and NGS in all 6 cases with the amplification of 5′ end of *EWSR1* locus (Figure 3). Six cases (40%, 6/15) with extra copy of *EWSR1* locus were from the femur. Four cases (67%, 4/6) showing EWSR1 amplification of 5′ end of the *EWSR1* locus were from tumours in the femur. No specific anatomical site was identified in the 8 cases with deletion signal pattern.

19 LGFMS were retrieved for evaluation using the FUS break-apart probe. The average age of LGFMS patients was 44 years (range 4–76 years) with a 1.4:1 male to female ratio. 68% (13/19) of the patients were >40 years old at the time of diagnosis. The most common sites of occurrence were the trunk (42%, 8/19) and proximal extremity (26%, 5/19). Among LGFMS, 32% (6/19) cases demonstrated abnormal signal patterns of extra copies with 2–4 yellow and extra copy of 5′ end of *FUS* locus with 1–2 green signals (Table 2). The *FUS::CREB3L2* fusion gene was detected in 4 cases by RT-PCR and not detected in 2 cases. No deletion or amplification of *FUS* locus was detected in 19 LGFMS detected.

Regarding the *USP6* gene rearrangement in 209 NF, the average age of NF patients was 37 years (range 3–81 years) with a 1.1:1 male to female ratio. The most common sites of occurrence were upper extremities (32%, 67/209), followed by trunk (21%, 44/209), head (11%, 24/209) and neck (10%, 20/209). 18 (9%) of 209 patient samples showed abnormal signal patterns of deletion (16 cases) with patterns of 1 yellow and 1 red signal (15 cases) or 1 yellow and 1 green signal (1 case). 2 cases showed extra copies using the USP6 break-apart probe, with 2 yellow and 1 green signal in a case, and 2 yellow and 1 to 4 red signals in another case. Among these 18 cases with abnormal signal patterns, the *MYH9::USP6* fusion gene was identified by RT-PCR and NGS in 11 cases (61%). *FRMD6::USP6* fusion gene was found by NGS in 1 case which had extra copy with 2 yellow and 1 to 4 red signals. *MYH9::USP6* fusion gene was not detected in 5 cases by RT-PCR. One case had no remaining material available for further testing.

207 SS were analysed using the SS18 break-apart probe, the average age of SS patients was 43 years (range 8–91 years) with a 1.1 male to female ratio. 67% (57/207) of the patients were below the age of 50 years. The most common sites of occurrence were extremities (65%, 134/207), followed by trunk (20%, 42/207), head and neck (4%, 9/207). 17 cases (8%) showed abnormal signal patterns of deletion of 3′ end of the *SS18* locus (8 cases) and extra copies of the *SS18* locus (9 cases). No amplification of *SS18* locus was identified. Among these 17 cases, *SS18::SSX1* and *SS18::SSX2* fusion genes were found in 13 cases (76%) and 3 cases (18%) by RT-PCR or NGS, respectively.

## Discussion

Current techniques such as panel NGS testing and WGS have advanced the classification of bone and soft tissue tumours and enabled a more comprehensive understanding of disease pathogenesis [1]. However, these techniques require specialized equipment, trained bioinformaticians and clinical scientists, and high quality material with high levels of tumour purity. Moreover, the turnaround times are slower than traditional techniques. RT-PCR is a relatively simple, and sensitive molecular assay to detect specific known fusion genes. However, RT-PCR may not be able to detect novel fusion genes due to variability in the transcriptome and variations of translocation partners. Ewing sarcoma with *EWSR1* gene rearrangement is presented as a prototypical example [13]. The breakpoints are variable in the introns of the *EWSR1* gene. The multiple fusion partners of *EWSR1* gene associated with Ewing sarcoma are *FLI1*, *ERG*, *ETV1*, *ETV4*, *FEV* and *ZSG*. Interphase FISH is rapid and can serve as a first line assay to detect the gene rearrangement in the routine pathological diagnostic workup of bone and soft tissue tumours. Fusion probes are used to support clinical diagnoses by detecting specific fusion genes when the abnormal signal patterns are observed by break-apart probes. However, the range of commercially available fusion probes is relatively less common and the signal patterns detected by break-apart probes are much more easily interpreted than those of fusion probes.

The frequency of abnormal signal patterns of break-apart probes in sarcomas are largely undescribed in the literature but poses a dilemma for clinical scientists. Papp, et al summarized 301 cases of soft tissue sarcomas with unusual signal patterns [6]. They found that 14% of sarcomas including ARMS, CCS, Ewing sarcoma, myxoid liposarcoma, LGFMS, NF and SS had unusual signal patterns. They considered deletion (1 yellow and 1 red/green), extra copy (2 yellow and 1-2 red or 1-2 green) and amplification as atypical signal patterns. However, the cases with a break-apart signal plus 1 additional yellow or 1 red signal were also considered as atypical signal patterns which are different from our classification of abnormal signal patterns. We classified the deletion, extra copy and amplification as three different types of abnormal signal patterns. If the cases showed a typical separated green and red signal regardless of additional few yellow or few red or few green signals, we considered these as positive gene rearrangements rather than the abnormal signal patterns.

In our present study, we retrospectively reviewed the FISH results from our routine diagnostic service using break-apart probes from 1,087 patient samples. In total, 80 (7%) of the 1,087 tumours had abnormal signal patterns (Table 2). From the soft tissue sarcomas cohort, we found abnormal patterns in alveolar rhabdomyosarcoma. A recurrent t (2; 13) (q35; q14)*PAX3::FOXO1* or t (1; 13) (p36; q14)*PAX7::FOXO1* chromosomal translocations are associated with alveolar rhabdomyosarcoma [1]. *PAX3::FOXO1* fusion gene is detected in 85% of alveolar rhabdomyosarcoma, while *PAX7::FOXO1* fusion gene is seen in 10% (Table 1). Abnormal signal patterns of *FOXO1* amplification in alveolar rhabdomyosarcoma is a known common phenomenon [1, 6]. 9 (10%, 9/94) cases of alveolar rhabdomyosarcoma showed amplification of 3′ end of the *FOXO1* locus in our study. To confirm whether these cases harboured *PAX3::FOXO1* or *PAX7::FOXO1* fusion gene, FISH with fusion probes and RT-PCR were perform. All of the cases with amplification of *FOXO1* locus carried either *PAX3::FOXO1* or *PAX7::FOXO1* fusion gene. *PAX7::FOXO1* fusion gene was detected in 6 cases. 3 cases showed *PAX3::FOXO1* fusion gene. These results were consistent with previous reports [14, 15].

The majority of translocations involving *EWSR1* gene in mesenchymal neoplasms are balanced translocations. Our results showed that the frequency of abnormal signal patterns of *EWSR1* in clear cell sarcoma is very low (2%, 1/61), which is consistent with a previous report [6]. Only one case with an extra copy of *EWSR1* locus was found. A novel *EWSR1::CREM* fusion gene was identified by WGS (Figure 6).

The Ewing sarcoma family of tumours is a group of malignant mesenchymal neoplasms characterized by characteristic *EWSR1* gene rearrangement. The most common fusion gene in Ewing sarcoma are *EWSR1::FLI1* (85%) followed by *EWSR1::ERG* (10%) (Table 1). Papp, et al reported one of 93 Ewing sarcoma showed two fusion and one extra red signal [6]. Our results showed that the abnormal signal patterns in Ewing sarcoma were deletions (2%, 8/491) and extra copies (3%, 15/491) of *EWSR1* locus. Among the 8 cases with a deletion pattern, *EWSR1::FLI1* was detected in 5 cases. *EWSR1::ERG* was found in 2 cases. The mechanism underpinning the deletion pattern is unknown and further studies are required to investigate the potential causes.

*EWSR1::NFATC2* sarcoma is a rare type of sarcoma, first reported by Szuhai et al [16]. This tumour exhibits characteristic clinical and histological features [16–18]. Unlike classical Ewing sarcoma, this tumour displays a characteristic FISH pattern with amplification at the 5′ end of the *EWSR1* locus [17, 18] This is due to a genomic rearrangement that fuses the N-terminal transactivation domain of EWSR1 to the C-terminal DNA binding domain of NFATC2, and this fusion is often accompanied by amplification of the 5′ end of the *EWSR1* locus. In the literature, this tumour is predominantly located in long bones, nearly half of cases were from the femur [1, 17]. Our results showed 4 of 6 cases were from femur. *EWSR1::NFATC2* fusion gene was detected by FISH and NGS in all 6 cases with the amplification of 5′ end of the *EWSR1* locus. These results support previous reports [1, 17, 18].

t (7; 16) (q34; p11)*FUS::CREB3L2* is the most common chromosome translocation in LGFMS (90%) [19]. 25% of cases harbour supernumerary ring chromosomes 7 and 16 [20, 21]. There are only a few published papers about abnormal signal patterns of break-apart FUS rearrangement in LGFMS [6, 20, 21]. Bartuma et al identified *FUS::CREB3L2* fusion gene in supernumerary ring chromosomes in a case of LGFMS [20]. The authors suggested that an unbalanced rearrangement of FUS with supernumerary ring chromosome explained the extra copy of *FUS* locus. Papp et al reported 4 of 6 cases (67%) LGFMS with two yellow and one or two isolated green signals [6]. However, no further evidence of *FUS::CREB3L2 or FUS* related fusion gene was investigated by RT-PCR or NGS in these four cases. Our study showed extra copy of 5′ end of the *FUS* locus as the sole abnormal signal pattern and was detected in 32% (6/19) of cases. *FUS::CREB3L2* fusion gene was found in 67% (4/6) of cases by RT-PCR.

Chen et al first reported the deletion of *USP6* locus in 1 of 8 cases of nodular fasciitis with *MYH9::USP6* fusion gene using FISH and RT-PCR [22]. An extra copy of the USP6 locus was detected in 1 of 6 cases of nodular fasciitis by Papp et al [6]. In our study, 9% (18/209) showed either deletion (16) or an extra copy (2) of *USP6* locus. *MYH9::USP6* fusion gene was detected in 12 cases by RT-PCR or NGS. A novel *FRMD6::USP6* fusion gene was identified in a case with an extra copy of *USP6* locus.

Synovial sarcoma harbours a pathognomonic t (X; 18) translocation resulting in either *SS18::SSX1*, *SS18::SSX2* or *SS18::SSX4* fusion genes [1]. *SS18::SSX1* is the most common fusion gene (90%) followed by *SS18::SSX2* (10%). *SS18::SSX4* is very rare. Amary et al first displayed deletion signals in 4 of 101 cases of synovial sarcoma by FISH *SS18* break-apart probes, thereby highlighting how these unusual patterns can cause uncertainty in the interpretation of FISH results and subsequent challenges in diagnosis [5]. *SS18::SSX* fusion gene was detected by RT-PCR in all 4 cases. Another study by Papp et al reported 6 of 89 synovial sarcomas showed abnormal signal patterns of the *SS18::SSX1* fusion gene using the TriCheck fusion probe and Real-Time PCR [6]. The authors suggested the interpretation of equivocal break-apart FISH results can be validated by FISH using fusion probes and RT-PCR [5]. In our study, abnormal signal patterns were found in 17 cases including 8 cases with deletion and 9 cases with extra copies. *SS18::SSX1* (13 cases) and *SS18::SSX2* (3 cases) were detected by RT-PCR and NGS in all cases except 1 case without material.

In total, 80 of 1,087 cases showed abnormal signal patterns in bone and soft tissue tumours using break-apart FISH probes. The specific fusion genes were detected by orthogonal methods including FISH with fusion probes, RT-PCR, NGS or WGS in 70 of 77 cases with material available. No specific fusion genes were detected by RT-PCR in 7 cases which could be due to either alternate fusion transcript breakpoints or alternative fusion partner genes that are not covered by primers for the RT-PCR assay.

The deletion signal pattern of the gene does not always have the rearrangement of the gene.

## Conclusion

This is a large, retrospective cohort from a specialist bone and soft tissue unit with systematic review of the interpretation of break-apart probe signals in bone and soft tissue tumours. In total, 7% (80/1,087) cases show abnormal break-apart signal patterns. The interpretation of these abnormal signal patterns may be challenging and can lead to false negative results or misdiagnosis. Our retrospective analysis highlighted the frequency of abnormal signal patterns by disease type and demonstrated the need validation testing using orthogonal molecular techniques -e.g. FISH Fusion probes, RT-PCR, targeted RNA sequencing (RNAseq) or Whole Genome Sequencing (WGS).

## Data Availability

Datasets used in producing this manuscript will be made available upon request to the corresponding authors.
